# Proton-Based Stereotactic Ablative Radiotherapy in Early-Stage Non-Small-Cell Lung Cancer

**DOI:** 10.1155/2014/389048

**Published:** 2014-07-17

**Authors:** Jonathan D. Grant, Joe Y. Chang

**Affiliations:** Department of Radiation Oncology, The University of Texas M.D. Anderson Cancer Center, Houston, TX 77030, USA

## Abstract

Stereotactic ablative radiotherapy (SABR), a recent implementation in the practice of radiation oncology, has been shown to confer high rates of local control in the treatment of early stage non-small-cell lung cancer (NSCLC). This technique, which involves limited invasive procedures and reduced treatment intervals, offers definitive treatment for patients unable or unwilling to undergo an operation. The use of protons in SABR delivery confers the added physical advantage of normal tissue sparing due to the absence of collateral radiation dose delivered to regions distal to the target. This may translate into clinical benefit and a decreased risk of clinical toxicity in patients with nearby critical structures or limited pulmonary reserve. In this review, we present the rationale for proton-based SABR, principles relating to the delivery and planning of this modality, and a summary of published clinical studies.

## 1. Introduction

Non-small-cell lung cancer (NSCLC) in its early stage can be treated definitively with favorable outcomes. The standard for curative therapy has historically been surgery for those medically able to undergo a lobectomy. However, given the frequent coincident morbidities such as elderly age, cardiac disease, and poor pulmonary function influenced by tobacco use, a significant proportion of patients are not operative candidates. Developing technologies have introduced less-invasive methods of addressing early-stage NSCLC with curative intent, including advances in external beam radiotherapy.

## 2. Stereotactic Ablative Radiotherapy

Conventionally fractionated radiotherapy, applied for over a century, delivers a protracted dose over multiple daily treatments commonly given over 5–7 weeks. This fractionated approach allows nearby normal tissues, which receive collateral amounts of radiation, to undergo cellular and DNA damage repair, thus minimizing damage to surrounding critical structures. Unfortunately, however, conventional fractionation for lung cancers has been shown to offer limited local control [1]. As improving technology in radiation planning, delivery, and motion management has evolved, stereotactic ablative radiotherapy (SABR) has emerged as an effective treatment for early-stage NSCLC. SABR is defined as a “high dose of radiation to an extracranial target in the body using either a single dose or a small number of fractions” [2]. SABR relies on precise localization of the tumor and careful consideration of nearby critical structures to avoid high doses in the sensitive region.

Applied to peripherally located early stage tumors <5 cm in size, SABR has been shown to produce a local control rate of >90%, with a low incidence of acute and long-term side effects [3]. The prescription dose strength of various radiotherapy fractionation schedules are often compared through 2-Gray biologically dose equivalent (BED) calculations. Studies have indicated that a BED of >100 Gray equivalents (GyE) is correlated with improved local control and survival [4, 5].

SABR was initially developed using photon-based radiotherapy, consisting of high-energy X-rays. Advantages of photon SABR include widespread availability, mature clinical experience, and good outcomes in appropriately selected patients. The application of protons in delivering SABR to early-stage NSCLC has emerged as a tool which may be able to reduce the risk of toxicity in patients with complicated presentations [6]. The advantage of proton therapy hinges on its ability to minimize dose to normal tissues distal to the tumor. Thus, potential clinical benefit may be offered to patients with limited pulmonary reserve, tumors in close geometric proximity to critical normal structures, or in patients who have received prior thoracic radiation [7, 8]. In these cases, reducing radiation damage to normal tissues is an absolute priority. The physical properties of protons, along with a discussion of technical issues related to delivery, planning considerations, and published clinical studies, will be presented herein.

## 3. Physical and Biologic Properties of Proton Therapy

Proton therapy is the most widespread application of charged particles for treatment of tumors in the body. Carbon ion therapy, used in several centers in Europe and Asia, will not be addressed in this review. Proton therapy was initially developed and clinically implemented in the mid-twentieth century, but it was limited to the treatment of ocular and intracranial tumors. These locations present minimal motion challenges and high fidelity with planning calculations. With improving technologies over the last several decades, the application of proton therapy has expanded to tumors throughout the entire body. Over 30 proton beam facilities are in operation worldwide, with many more being under construction and being expected to open over the next several years.

**Figure 1:**
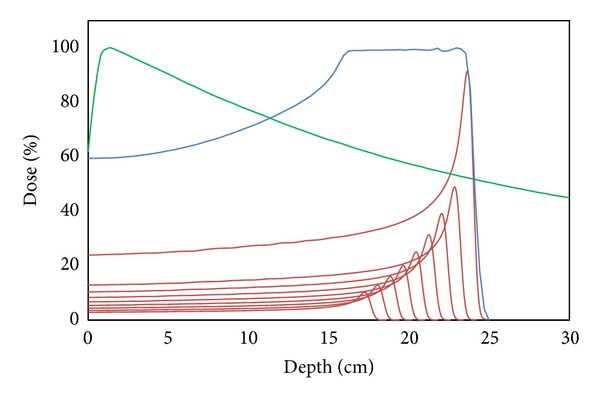
Demonstration of the Bragg peak that is characteristic of proton therapy. Individual proton beam energies are represented by the multiple red curves, with higher energies depositing their maximum energy at an increased depth. The summation of these individual beams is represented by the blue curve, known as the spread-out Bragg peak (SOBP). This SOBP is calculated and delivered such that the full depth of the target receives this maximum radiation dose. The sharp falloff following the Bragg peak allows tissues distal to the target to be spared. The green curve represents the dose deposition profile of X-ray therapy. In contrast to proton therapy, the maximum dose is deposited within several centimeters of tissue penetration and distal tissues receive a gradually decreasing amount of radiation exposure.

The defining characteristic of proton therapy is the “Bragg peak,” a description of the high-energy dose deposition over a small distance at the end of the proton range (Figure 1). Proximal to this peak is a low entrance dose profile, within minimal or no radiation dose to distal tissues. The range of a proton is inversely proportional to the density of the tissue. In contrast, photon-based radiotherapy delivers its peak energy within several centimeters of tissue penetration, followed by a gradual dose decline, resulting in a penetration of X-rays through the body with a larger area of tissue exposed to collateral radiation (Figure 1). This improved selectivity in radiation dose delivery may help reduce normal tissue toxicity, allow for higher dose delivery to tumor, and reduce the chance of secondary late malignancies.

Biologic differences also exist between protons and photons, with regard to how these different types of radiation interact with living cells. The nature of these effects and implications on tumor control and normal tissue toxicity is largely unknown at the present time and represents an area of growing research. Protons have been found to have a higher linear energy transfer (LET) than photons, which quantifies the magnitude of DNA damage from reactive ions. This LET effect also increases near the distal end of the Bragg peak by about 5–10% [9]. These differences have been shown to have biologic effects, including a greater impact on paclitaxel-resistant cell lines and may translate into a greater effect on recurrent or persistent NSCLC [10]. Multiple researches have reported larger foci of DNA repair as measured by immunohistochemical staining of H2AX foci following proton therapy when compared to photons [11, 12]. Protons have been shown to produce more DNA fragments [13], methylation of DNA [14], activation of free radicals [15], modulation of gene expression [16], and apoptotic activity [17]. The implication of these effects and clinical application to leverage the differences that proton therapy offers largely remain to be elucidated.

## 4. Proton Therapy Delivery

A proton is obtained by stripping a hydrogen atom of its electron, resulting in a positively charged hydrogen ion. These ions are then accelerated to a typical energy of 70–250 mega electron volts (MeV) by a synchrotron or cyclotron. These protons are then delivered to a patient who is typically lying on a treatment table in a shielded room and directed to the appropriate depth in the body through either a “passively scattered” or “beam scanning” technique. Passively scattered proton therapy (PSPT) consists of a shaped proton beam delivered instantaneously to the patient. The lateral boundaries of the field shape are defined by passing the beam through an aperture cut out from heavy metal. A custom-produced compensator is inserted into the beam path to modulate the proton depth at different points across the field. Finally, a rotating range shifter distributes the Bragg peak over the depth of the target, creating what is known as the spread-out Bragg peak (SOBP). PSPT is typically delivered with 1–4 beams in order to reduce planning uncertainties and restrict the broad deposition of radiation in the lungs [18].

In contrast, scanning beam (or intensity-modulated proton therapy, IMPT) consists of sequential targeting of 300–600 spots in a voxel-like array. These spots can measure 3–25 mm in size and are also known as pencil beams. The computational resources needed for this technique are significant, as each pencil beam is simultaneously optimized with inversely planned objectives. This approach allows complex targets to be treated with greater conformality, because the depth and shape of the Bragg peak are altered along the path of each beam axis. This potential for greater conformality is a double-edged sword however, with tighter margins conferring the potential for missing the target if a planning calculation differs from the actual trajectory of the beam or if the tumor moves. Technological advances to address the challenges inherent in this emerging technology and simplify the application of this emerging modality are under development [19, 20]. A technology fundamental to photon therapy that is present in newer proton centers is on-board CT scans for verification of patient positioning at the time of treatment. The first generation of proton centers was equipped with 2-dimensional verification X-rays only for treatment verification, a limitation that will be met with newer centers and equipment upgrades. Further advances may be realized with improved accuracy in the images used to plan proton therapy. Currently, the relative proton stopping is determined from calculations based on the Hounsfield unit measurements of the planning CT scan. These calibrations introduce systematic error and are a source of range uncertainty, especially in the setting of image artifact [21]. The use of proton-based computed tomography is being developed to create an accurate electron density map for calculation of proton stopping power in the planning process [22].

## 5. Motion Management and Planning Techniques

Limiting motion and careful accounting for it in treatment planning is a hallmark of SABR. Consistency between the planning position and delivery position of the patient is especially crucial for proton-based SABR, as the specific density of tissue that each charged particle traverses determines its depth dose. If this path length is significantly altered with motion or setup variability, target miss or overtreatment of normal tissues could occur. This becomes especially important in the thorax, where tissue heterogeneity also increased uncertainties. Techniques to limit tumor motion and account for it in planning process are similar to those used in photon therapy. These include planning CT scans that characterize the path of the tumor over the entire tumor cycle (4DCT), external body frames and internal reference fiducials, compression devices, and respiratory gating systems to limit motion or synchronized radiation delivery with the breathing cycle.

Motion management is particularly important for IMPT, because the target is radiated sequentially. Intrafraction motion could thus potentially result in hot and cold spots within the tumor. To mitigate this interference between the scanning patterns and intrafractional movement, the tumor may be covered with multiple beam scans. In addition, the use of larger pencil-beam spots is more resistant to tumor motion degradation [6, 19, 23]. Adjustment for planning uncertainties in the case of PSPT is achieved using a smearing technique, in which the compensator is modified to maintain tumor coverage in the presence of small range uncertainties, at the sacrifice of some conformality.

**Figure 2:**
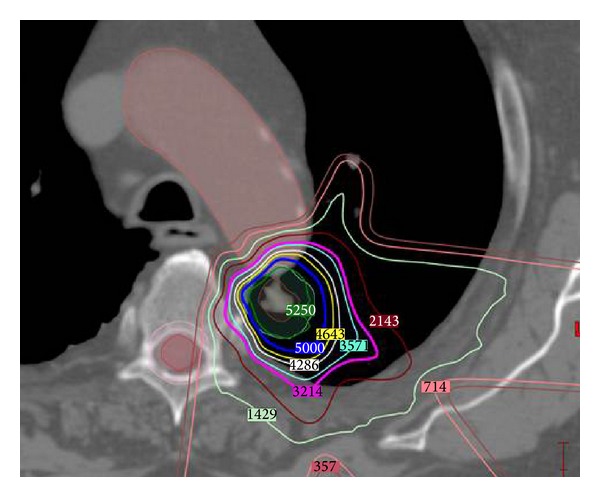
Radiation treatment plan illustrating the dosimetric benefits of proton therapy in a patient with tumor near critical central structures. Prescribed tumor dose is 50 GyE in 4 fractions, with isodose line numbers displayed in units of cGyE. Significant radiation sparing of the aorta, esophagus, and lung is achieved due to the steep dose falloff of protons, while achieving appropriate target coverage for tumor cell kill. Beam angles are selected to traverse a minimal amount of lung tissue. Range differences based on the density heterogeneity of tissue traversed can be appreciated at the anterior aspect of the plan, where a peak of dose is deposited in normal lung. Careful attention must be paid to these dose variations and areas of range uncertainty such that dose tolerance of critical structures is not exceeded.

Target volume expansions must be considered for each separate beam direction and the unique anatomic path that these protons will traverse [24]. Due to the uncertainty of tumor motion, proton range, and the lack of volumetric image guidance in most current proton centers, generous margin is typically added to ensure target coverage. This margin can be significantly reduced when fiducial tracking implants and respiratory gating are used, as on-board volumetric image guidance is introduced, a better understanding of proton range uncertainty is gained, and treatment planning is optimized, particularly for IMPT. In light of the physical properties and planning process of proton therapy, the potential clinical benefit derived from proton SABR is contingent upon a variety of patient-specific factors. These include the geometric location of critical normal structures in relation to the target, the beam angles utilized, the uncertainties associated with each beam path, and the likelihood of these reductions in normal tissue to have meaningful clinical impact in the context of the overall clinical scenario (Figure 2).

## 6. Dosimetric and Clinical Studies

A number of dosimetric studies investigating the differences in radiation dose delivered to normal tissues for proton versus photon-based SABR have been reported [6, 25–29]. These studies show the potential reduction in radiation dose to the lung, esophagus, brachial plexus, chest wall, and heart with the use of protons when compared with photon-based SABR, as shown in Table 1.

A phase II randomized clinical trial comparing proton versus photon-based SABR for centrally located or recurrent lung parenchymal early-stage NSCLC is currently ongoing in our institution. Several single-institution retrospective studies have been published and are summarized in Table 2. Given the variability in fractionation regimens and varying definitions of what constitutes SABR, we have focused on studies that included fraction sizes greater than 4 Gy.

Loma Linda University has the longest-term experience with proton SABR, as reported by Bush et al., where treatment was escalated from 51 GyE to 70 GyE in 10 fractions [30]. Local control and survival were found to improve with escalated doses. Overall survival at 5 years was 18%, 32%, and 51% at 51 GyE, 60 GyE, and 70 GyE, respectively; however this is also subject to potential biases in overall therapeutic improvements in later time periods. Tumors greater than 5 cm were associated with worse local control. There were no cases of radiation pneumonitis, suggesting room for increased dose delivery to improve outcomes. PTV coverage was 3–5 mm. As is the case here and all of the following studies, immobilization was achieved with a cradle and respiratory management was achieved with respiratory gating, kV imaging, and 4DCT in selected patients treated after 2005.

Another experience from Hata et al. included 21 patients treated with doses escalated from 50 GyE to 60 GyE in 10 fractions, as in the previously described study [31]. A 5 mm margin in the plane perpendicular to the beam axis and a 5 mm caudal margin were added for respiration movement. Only one local recurrence was reported at 2 years (95% local control), with an overall survival of 74%.

Nakayama et al. treated 55 patients with 58 tumors to either 66 GyE in 10 fractions or a more protracted course for central tumors of 72.6 GyE in 22 fractions for central tumors [32]. This fractionated course allows for more sublethal repair of critical centrally located normal tissues. Two patients developed grade 3 pneumonitis. Planning margins included a 5 mm PTV for setup uncertainty, and an additional 5 mm margin in the caudal direction.

Nihei et al. reported 37 patients with T1 and T2 tumors below 5 cm, treated with a total dose of 70–94 GyE in 20 fractions [33]. More than half of the patients had T2 tumors, though tumor size was limited to <5 cm. Two-year local control and survival were 98% and 84%, respectively. Although acute toxicities were minimal, six patients experienced late pulmonary toxicity. These late toxicities may have resulted from an excess volume of normal lung irradiated due to tumor shrinkage over the 4-week course. The importance of adaptive replanning in this context was illustrated by Chang et al., who found that 45% of patients with early-stage NSCLC treated over 7 weeks with protons therapy benefited from a repeat simulation and radiation plan due to tumor shrinkage [34]. As in the prior study, PTV margin was 5 mm, with an additional 5 mm margin used caudally.

Iwata et al. and Fujii et al. have published three series of patients treated with proton and carbon ion therapy for early stage NSCLC. The first report included patients with T1-T2 tumors treated with 80 GyE in 20 fractions or 60 GyE in 10 fractions in the case of protons, which comprised the majority of patients [35]. Three-year local control and survival were 83% and 60%, respectively, for patients treated with 60 GyE, and 81% and 90%, respectively, for patients treated with 80 GyE. The second report analyzed a subset of 70 patients with T2 disease only, 43 of whom were treated with proton therapy with the addition of several different fractionation schedules [36]. Four-year local control and overall survival were 75% and 78%, respectively, and no significant differences were seen between the T2a and T2b groups. Minimal toxicity was observed in patients with centrally located tumors, highlighting the potential benefit of protons for tumors with clinically challenging locations and larger size. The most recent report included 70 patients and reported a 3-year local control of 81% with overall survival at 3 years being 72% [37]. Planning margins for these studies included a 5 mm PTV, with a 1–4 mm internal margin depending on the stability of the respiration.

Westover et al. published from the most recent cohort of patients, with 20 early-stage NSCLC tumors treated with 42–50 GyE in 3 to 5 fractions using PSPT [38]. Most of these tumors were located in a favorable position in the upper lung where breathing motion has a limited impact. Two-year LC and OS were 100% and 64%, respectively. One patient developed grade 3 pneumonitis which resolved with steroids. A 5 mm PTV was added, additional unspecified margin for respiratory motion, as well as 3.5% of the proximal and distal ranges plus 2 mm to the proximal and distal portions of the GTV.

The above studies indicate a trend towards improved local control with more recent studies, highlighting the improving nature of this emerging technology. Recognizing the limitations of meta-analysis given the heterogeneous protocols, Grutters et al. reported the 5-year pooled overall survivals for proton therapy, carbon ion therapy, photon SABR, and conventionally-fractionated radiation to be 40%, 42%, 42%, and 20%, respectively [39].

## 7. Conclusions

As the availability of proton therapy expands and the experience matures, improvements will continue to be made in the implementation of this technology. Emerging techniques such as IMPT and on-board volumetric image guidance will continue to reduce uncertainties and refine conformality [40–44]. The unique properties of protons to spare tissue distal to the target position it as a valuable tool available to the radiation oncologist for patients with critical structures near tumor, larger T2 tumors, or limited pulmonary reserve. Efforts to produce randomized data are ongoing to directly compare outcomes between photon and proton SABR. With existing clinical data, proton SABR is an effective treatment option for patients with early-stage NSCLC who may derive benefit from maximal normal tissue sparing.

## Figures and Tables

**Table 1:** Dosimetric reduction in normal tissue radiation for proton- versus photon-based SABR plans.

Author, reference	Number of plans compared (*n*)	Total dose, GyE (dose per fraction)	Lung reduction from protons	Esophagus reduction from protons
Hoppe et al., [25]	16	48 (12)	Mean dose 2.2 GyE *V*5, 10.4%∗ *V*10, 6.4% *V*20, 2.1%	

Seco et al., [26]	20	42 (14)	*V*5, 37.8%	Maximum dose, 68%

Georg et al., [27]	36	45 (15)	*V*20, PSPT, 7–9% *V*20, IMPT, >10%	

Register et al., [6]	45	50 (12.5)	Mean dose, 50%	

Kadoya et al., [29]	21	66 (6.6)	Mean dose 2.8 GyE *V*5, 18.8% *V*10, 10.4% *V*20, 2.3%	

*Vx: percentage of structure volume receiving ≥*X* GyE. PSPT: passively scattered proton therapy. IMPT: intensity-modulated proton therapy.

**Table 2:** Summary of clinical data for proton-based stereotactic ablative and hypofractionated radiotherapy.

Author, reference	Years	Number of cases (T1/T2)	Total dose, GyE (dose per fraction)	Local control	Overall survival	Toxicity grade ≥3
Bush et al., [30]	Unknown	111 (47/64)	51/60/70 (5.1/6/7)	4 yr, 45/75/86/91%∗	4 yr, 18/32/51%^†^	None
Hata et al., [31]	2002–2005	21 (11/10)	50–60 (5-6)	2 yr, 95%	2 yr, 74%	RP 4%
Nakayama et al., [32]	2001–2008	58 (30/28)	66-peripheral/72.6-central (6.6/3.3)	2 yr, 97%	2 yr, 98%	RP 4%
Nihei et al., [33]	1999–2003	37 (17/20)	70–94 (3.5–4.9)	2 yr, 98%	2 yr 84%	RP 8%
Iwata et al., [35]	2003–2007	57 (27/30)	60/80 (6/4)	3 yr, 83/81%^‡^	3 yr, 60/90%^‡^	RP 2%, dermatitis 5%
Iwata et al., [36]	2003–2009	43 (0/43)	60/66/70.2/80 (6/6.6/2.7/4)	3 yr, 75%	3 yr, 78%	RP 3%, dermatitis 7%^§^
Fujii et al., [37]	2003–2009	70 (36/34)	60/80 (6/4)	3 yr, 81%	3 yr, 72%	RP 0%, dermatitis 4%, rib fracture 1%^||^
Westover et al., [38]	2008–2010	20 (18/2)	42–50 (10–16)	2 yr, 100%	2 yr, 64%	RP 7%

*Reported for the following groups: (T2, 60 GyE)/(T2, 70 GyE)/(T1, 60 GyE)/(T1, 70 GyE), ^†^Reported for: 51 GyE/60 GyE/70 GyE. ^‡^Reported for 60/80 GyE. ^§^Toxicity data includes 27 combined patients treated with carbon ion therapy, which were not separated in the manuscript. ^||^Termed late toxicity, time period not defined. RP: radiation pneumonitis.
